# Discovery of Novel Indolealkylpiperazine Derivatives as Potent 5-HT_1A_ Receptor Agonists for the Potential Future Treatment of Depression

**DOI:** 10.3390/molecules25215078

**Published:** 2020-11-01

**Authors:** Chen Zhu, Xinwei Li, Weiqing Peng, Wei Fu

**Affiliations:** Department of Medicinal Chemistry, School of Pharmacy, Fudan University, 826 Zhangheng Road, Pudong New District, Shanghai 201203, China; 18211030013@fudan.edu.cn (C.Z.); 19211030070@fudan.edu.cn (X.L.); 15211030063@fudan.edu.cn (W.P.)

**Keywords:** 5-HT_1A_ receptor agonist, depression, drug design, molecular dynamics

## Abstract

Depression is a severe psychiatric disorder that affects over 100 million people worldwide. 5-HT_1A_ receptor agonists have been implicated in the treatment of a variety of central nervous system diseases, especially depression. In this study, based on **FW01**, a selective potent 5-HT_1A_R agonist discovered via dynamic pharmacophore-based virtual screening, a series of indolealkylpiperazine derivatives with a benzamide moiety were designed and synthesized by the modification of the amide tail group as well as indole head group of **FW01**. Among all tested compounds, **13m** displayed potent agonistic activity towards 5-HT_1A_R with an EC_50_ value of 1.01 nM. Molecular docking studies were performed to disclose the mechanism of its potent agonistic activity and high selectivity. Finally, the activation model of 5-HT_1A_R induced by **13m** was proposed.

## 1. Introduction

Depression is a severe psychiatric disorder that affects over 100 million people worldwide [1]. The main symptoms include low mood, lack of energy, sadness, insomnia, etc. [2]. Patients also suffer a high risk of suicide [3,4]. Though the etiology of depression is not clear, it is certain that genetic and psychological factors as well as environmental factors are all involved in the pathogenesis [5,6]. Selective serotonin reuptake inhibitors (SSRIs) and serotonin norepinephrine reuptake inhibitors (SNRIs) are common antidepressants clinically [7,8,9]. Though SSRIs are first-line drugs for the treatment of depression, they still have some drawbacks including delayed onset and limited efficacy [10,11]. Thus, drug discovery for new antidepressants with rapid onset and good efficacy is still urgent [12,13].

Serotonin (5-hydroxytryptamine, 5-HT), as an important neurotransmitter, is involved in multiple physiological and behavioral processes [14,15]. 5-HT mediates its physiological effects through at least 14 different receptor subtypes [16]. Among them, 5-HT_1A_ receptor (5-HT_1A_R) plays a significant role in mood regulation and has been a hot target for various central nervous system disorders, especially depression [17,18,19,20]. Medicines like gepirone, tandospirone and vilazodone acting as 5-HT_1A_R partial agonists are representative antidepressants clinically [21]. Meanwhile, the combination of 5-HT_1A_R agonists and SSRIs not only accelerate the onset of SSRIs but also improve the therapeutic effect [10]. Therefore, based on our previous research, we aimed at discovering selective 5-HT_1A_R agonists with novel scaffold with the eventual aim to treat depression.

## 2. Results and Discussion

### 2.1. Compound Design

**Figure 1:**
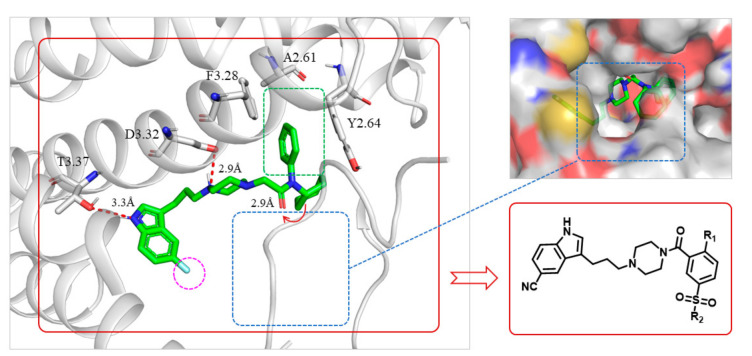
Predicted binding mode of **FW01** (green sticks) with 5-HT_1A_R (cartoon) and the design of indolealkylpiperazine derivatives. Ligands are shown as green sticks. The binding residues of 5-HT_1A_R are shown in gray. Hydrogen bond interactions are represented as dotted red lines. 5-HT_1A_R was obtained by homology modeling from the X-ray structure of 5-HT_1B_R (PDB code: 6G79).

In our previous study, a series of lead compounds as 5-HT_1A_R agonists with novel scaffolds were obtained through the combination of pharmacophore-based virtual screening and biological assay [22]. Among them, **FW01** (Figure 1) possessed high activity for 5-HT_1A_R (*K*i = 51.9 ± 16.4 nM), medium activity for 5-HT_2A_ receptor (5-HT_2A_R) (*K*i = 206.71 ± 7.46 nM) and low activity for dopamine D_2_ receptor (D_2_R) (*K*i = 2161.35 ± 25.55 nM). **FW01** was bound to 5-HT_1A_R via multiple interaction forces [22].

The docking predicted binding mode of **FW01** with 5-HT_1A_R (Figure 1) showed that the indole head group was inserted into the pocket formed by transmembrane helix 3 (TM3), TM5, TM6 and TM7 of 5-HT_1A_R. The NH group of the indole head formed hydrogen bonding with T3.37. The protonated nitrogen of piperazine participated in the salt bridge with D3.32. As for the amide tail group, the phenyl ring was inserted into the pocket formed by residues F3.28, Y2.64, and A6.21. However, there was an empty pocket formed by TM6 and TM7 which was not reached by **FW01**. Due to the presence of the upper pocket and the lower pocket formed by TM6 and TM7, the amide tail moiety of **FW01** was replaced with different 2,5-substituted benzamide moieties. As for the indole moiety, the development of marketed antidepressant drug vilazodone showed that a 5-CN indole moiety could enhance the 5-HT_1A_R affinity, which was also proved in our previous study [23,24]. Therefore, the 5-F indole moiety of **FW01** was replaced by 5-CN indole moiety to raise the agonistic activity of designed compounds towards 5-HT_1A_R.

Finally, a series of indolealkylpiperazine derivatives with a benzamide moiety were designed and synthesized. Their cellular function activities on dopamine and serotonin receptors were evaluated by Ultra Lance cAMP assay and Fluorometric Imaging Plate Reader (FLIPR) assay.

### 2.2. Chemical Synthesis

**Scheme 1:**
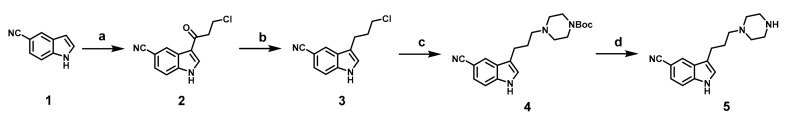
Synthesis of intermediate **5**. Reagents and conditions: (**a**) 3-chloropropionyl chloride, AlCl_3_, anhydrous DCM, r.t., overnight; (**b**) NaBH_4_, CF_3_COOH, anhydrous THF, 0 °C, 1 h; (**c**) Boc-piperazine, CH_3_CN, K_2_CO_3_, 75 °C, 36 h; (**d**) CF_3_COOH, DCM, r.t., 4 h.

Synthesis of intermediate **5** was shown in Scheme 1. Commercially available 5-cyanoindole was first acylated with 3-chloropropionyl chloride via Friedel-Crafts acylation to obtain **2**, which was reduced by NaBH_4_/CF_3_COOH system to give compound **3**. Compound **4**, which was obtained via the reaction of **3** and N-Boc-piperazine, was deprotected to give **5**.

**Scheme 2:**
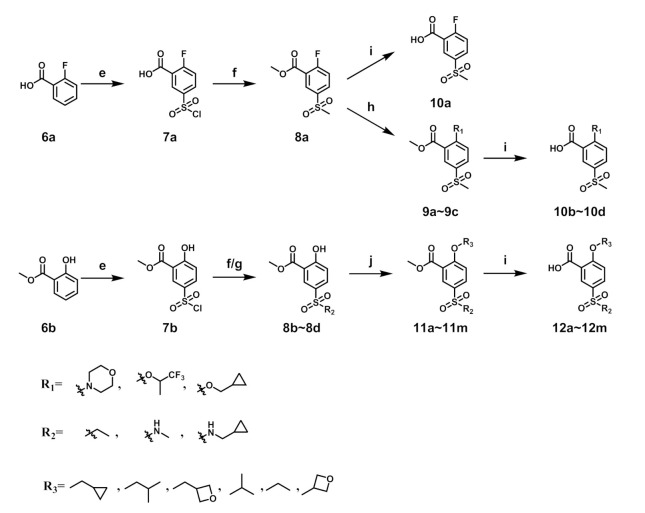
Synthesis of benzoic acid intermediates. Reagents and conditions: (**e**) HClSO_3_, 40 °C, 30 min; (**f**) i. Na_2_SO_3_, H_2_SO_4_, H_2_O, r.t., 2 h; ii. iodide alkanes, K_2_CO_3_, DMF, r.t., 3 h; (**g**) amine, DCM, r.t., 2 h; (**h**) corresponding reagent, K_2_CO_3_, DMF, 150 °C, 18 h; (**i**) LiOH·H_2_O, THF:H_2_O = 4:1, r.t., 5 h; (**j**) alcohol, PPh_3_, DBAD, THF, r.t., 6 h or 3-iodo-oxetane, Cs_2_CO_3_, DMF, 80 °C, 10 h.

Synthesis of the 5-sulfonyl benzoic acid was outlined in Scheme 2. Compound **7a** was obtained by the sulfonation reaction of **6a** and chlorosulfonic acid. **7a** reacted with sodium sulfite and then alkylated by iodomethane to give **8a**. **8a** was deprotected by lithium hydroxide to give **10a**. Synthesis of **10b**–**10d** was accomplished by reaction of **8a** and corresponding reagents and then deprotection by lithium hydroxide. For **8b**–**8d**, the starting material was methyl salicylate and the synthetic route was similar to **8a**. Compounds **11a**–**11m** were obtained by Mitsunobu reaction of **8b**–**8d** and corresponding alcohol or alkylation of **8b**–**8d** and 3-iodo-oxetane, which were then deprotected by lithium hydroxide to give compounds **12a**–**12m**.

**Scheme 3:**
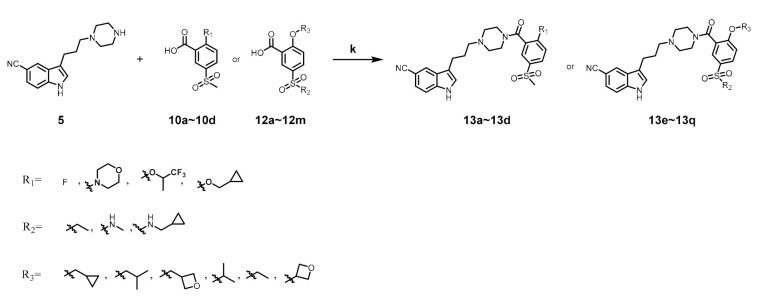
Synthesis of compounds **13a**–**13q**. Reagents and conditions: (**k**) EDC·HCl, HOBT, DIEA, DCM, r.t., 8 h.

As shown in Scheme 3, compounds **13a**–**13q** were obtained by condensation of **5** and benzoic acid intermediate. Structure of all compounds were confirmed by ESI-MS, HRMS, ^1^H NMR and ^13^C NMR.

### 2.3. In Vitro Biological Activity Evaluation

After these new derivatives were synthesized, they were all subjected to in vitro evaluation of functional activities towards 5-HT_1A_, 5-HT_2A_ and D_2_ receptors. As shown in Table 1, indolealkylpiperazine derivatives displayed varied selectivity for 5-HT_1A_R over D_2_R and 5-HT_2A_R. When R_2_ was methyl group, R_1_ was subjected to substituents of different size to examine the SAR. Compound **13a** with an F atom displayed reduced agonistic activity for 5-HT_1A_R. The introduction of morpholine group (**13b**) resulted in drastically decreased activity towards three receptors, indicating that large size group of R_1_ position was not compatible with these receptors. Replacing morpholine group with smaller size 1,1,1-trifluoro-2-propyl or cyclopropylmethoxy group afforded **13c** and **13d**, respectively. By comparing **13b**, they showed increased activity for 5-HT_1A_R and 5-HT_2A_R. We postulated that the cyclopropylmethoxy group extended into the hydrophobic pocket where the phenyl ring of **FW01** located, thus stabilizing the binding conformation of **13d** in the active pockets of 5-HT_1A_R and 5-HT_2A_R. Then, the ethyl, methylamino and cyclopropylmethylamino groups were employed to replace methyl of **13d** and compounds **13e**–**13g** were designed. When adopting the sulfonamide substituents (**13f** and **13g**), the activity for 5-HT_1A_R showed a remarkable elevation in comparison to compound **13a**–**13e**, presumably due to the less flexible nitrogen chain posing a limited conformation to form a more stable hydrophobic interaction.

Encouraged by the results above, we next extended our efforts to investigate the effect of substituents of position 2 of the phenyl ring on compounds **13f** and **13g**. Overall, compounds **13f**–**13q** showed selective agonistic activity for 5-HT_1A_R. Among these compounds, **13g** and **13m**–**13q** bearing cyclopropylmethylamino moiety displayed more potent activity for 5-HT_1A_R than their corresponding compounds **13f** and **13h**–**13l** bearing methylamino moiety, suggesting that cyclopropylmethylamino moiety was more favorable than methylamino moiety at position 2 as shown in Table 2. Compounds **13h** and **13m** bearing the isobutyl moiety were found to demonstrate the most potent agonistic activity for 5-HT_1A_R among methylamine and cyclopropylmethylamino moiety possessing compounds, respectively. Compound **13j** with isopropyl group was found to completely lose activity for 5-HT_1A_R, which was due to the isopropyl moiety of **13j** forming steric hindrance with 5-HT_1A_R. As for **13o** which showed potent activity for 5-HT_1A_R, the presence of the N-(cyclopropylmethyl)sulfamoyl moiety limited the conformation of the compound. By contrast, the introduction of a polar atom to the R_3_ group resulted in a decreased activity for 5-HT_1A_R by an order of magnitude, indicating that a polar group was unfavorable to the binding affinity towards 5-HT_1A_R (**13i**, **13l**, **13n**, **13q**).

### 2.4. Binding Mode

**Figure 2:**
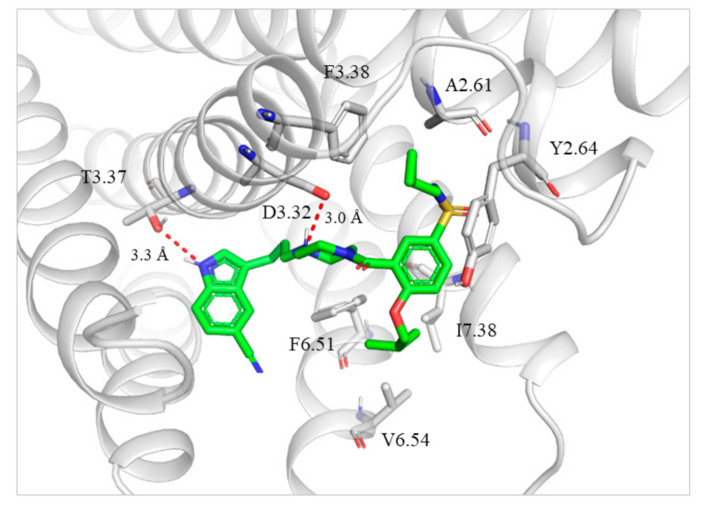
Binding mode of **13m** with 5-HT_1A_R. The ligand is shown as green sticks. 5-HT_1A_R and the surrounding residues are in gray. Hydrogen bond interactions are represented as dotted red lines.

We next studied the binding mode of **13m** with agonistic conformation of 5-HT_1A_R. We used the GOLD 5.1 program to dock **13m** into the binding site of the 5-HT_1A_R which was obtained by homology modeling from the X-ray structure of 5-HT_1B_ receptor (PDB code: 6G79) [25]. The results of molecular modeling were shown in Figure 2. The protonated nitrogen atom of **13m** formed a strong salt bridge with D3.32 in 5HT_1A_R. The NH group of the indole ring formed hydrogen bonding with T3.37. The N-(cyclopropylmethyl)sulfamoyl moiety interacted with A2.61, Y2.64 and F3.28 through hydrophobic packing, which was in accordance with the binding mode of **FW01** with 5-HT_1A_R. The isobutoxy group was inserted into another hydrophobic pocket formed by F6.51, V6.54 and I7.38.

**Figure 3:**
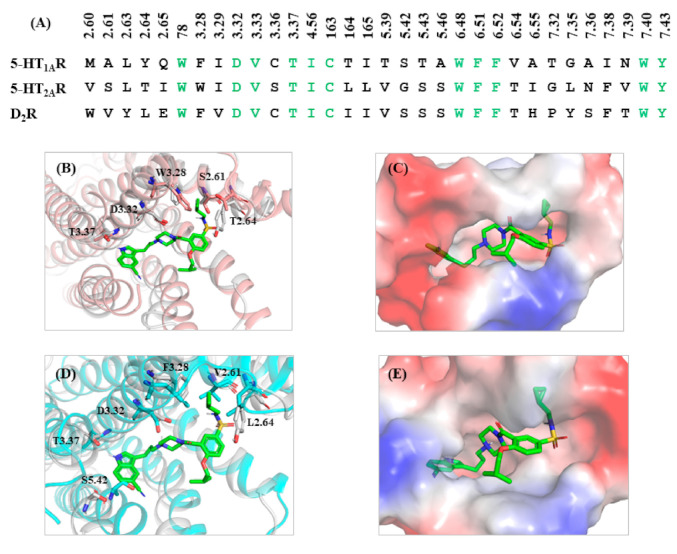
(**A**) Sequences of 5-HT_1A_R, 5-HT_2A_R, and D_2_R at the binding site. (**B**) The superimposition of 5-HT_2A_R with **13m**-bound 5-HT_1A_R. (**C**) Electrostatic potential energy diagram of **13m** and 5-HT_2A_R at the binding site. (**D**) The superimposition of D_2_R with **13m**-bound 5-HT_1A_R. (**E**) Electrostatic potential energy diagram of **13m** and D_2_R at the binding site. The ligand is shown as green sticks. 5-HT_1A_R and the surrounding residues are in gray. 5-HT_2A_R and the surrounding residues are in pink. D_2_R and the surrounding residues are in blue.

To explain the high selectivity of **13m** toward 5-HT_1A_R, the sequences of 5-HT_1A_R, 5-HT_2A_R and D_2_R at the binding site were aligned. As shown in Figure 3A, the residues of the active site in three receptors differed greatly and thus the conformations of the three receptors at the binding site were different. The 3D structures of **13m**-bounded 5-HT_1A_R and 5-HT_2A_R were superimposed to explain the selectivity of **13m** for 5-HT_1A_R and 5-HT_2A_R. The PDB code of 5-HT_2A_R was 6A93 [26]. As shown in Figure 3B, compared with 5-HT_1A_R, the amino acid residues in the hydrophobic pocket of 5-HT_2A_R stretched out further. Thus the N-(cyclopropylmethyl)sulfamoyl of **13m** collapsed with W3.28 in the active pocket of 5-HT_2A_R, which is obvious to be observed in Figure 3C. It accounted for the reduced affinity of **13m** for 5-HT_2A_R. The same phenomenon was found in the case of D_2_R when superposing 5-HT_1A_R and D_2_R (Figure 3D). The PDB code of D_2_R was 6CM4 [27]. The amino acid residues in the active pocket of D_2_R stretched out further than those in the active pocket of 5HT_1A_R. Thus the indole head and the N-(cyclopropylmethyl)sulfamoyl of **13m** collapsed with S5.42 and L2.64 in the active pocket of D_2_R. This explains why **13m** showed high selectivity toward 5-HT_1A_R over 5-HT_2A_R and D_2_R.

### 2.5. 13m Induced Activation Mechanism of 5-HT_1A_R

Molecular dynamic simulations were carried out to explore the activation process of 5-HT_1A_R by **13m**. Two systems, **13m** bound to the active-state 5-HT_1A_R and the inactive-state 5-HT_1A_R without ligand (apo), were performed for 200 ns simulation, respectively. The inactive-state 5-HT_1A_R was modelled from the X-ray structure of 5-HT_1B_R (PDB code: 5V54). The Gromos conformational cluster analysis was used to obtain the representative structures of two systems in the course of simulation.

**Figure 4:**
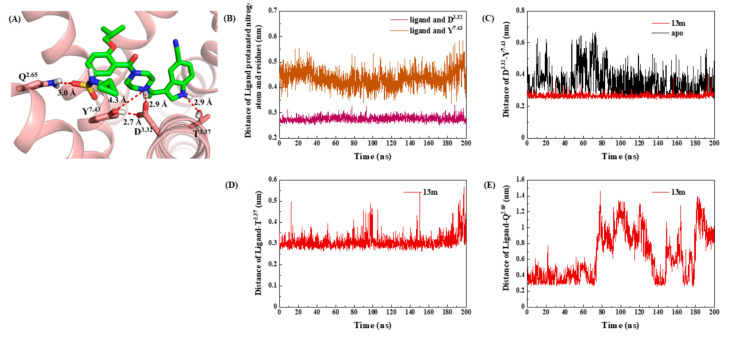
(**A**) The interaction between **13m** and D3.32, Y7.43, Q2.65 and T3.37. The ligand is shown as green sticks. 5-HT_1A_R and the surrounding residues are in pink. Hydrogen bond interactions are represented as dotted red lines. (**B**) Distance of the ligand with D3.32 and Y7.43. (**C**) Distance between D3.32 and Y7.43. (**D**) Distance between ligand and T3.37. (**E**) The interaction between ligand and Q2.65 in **13m**-5-HT_1A_R system.

In **13m**-5-HT_1A_R system, a conserved hydrogen bond was formed between the NH group of the indole ring in **13m** and the oxygen atom of hydroxyl in T3.37 during the simulation (Figure 4A,D). At the same time, the protonated nitrogen atom in **13m** maintained a stable hydrogen bond with the carbonyl oxygen of D3.32 and a relatively weak interaction with the hydroxyl oxygen of Y7.43 (Figure 4B). Therefore, the interaction between D3.32 and Y7.43 (also called 3-7 lock) stayed unbroken in **13m**-5-HT_1A_R system but experienced big fluctuation in apo system (Figure 4C). This phenomenon was in agreement with the previous study of our group [28]. The oxygen atom on the sulfonyl group of **13m** formed a hydrogen bond with Q2.65 initially, but it fluctuates greatly due to the swing of the molecular tail after 80 ns (Figure 4E).

**Figure 5:**
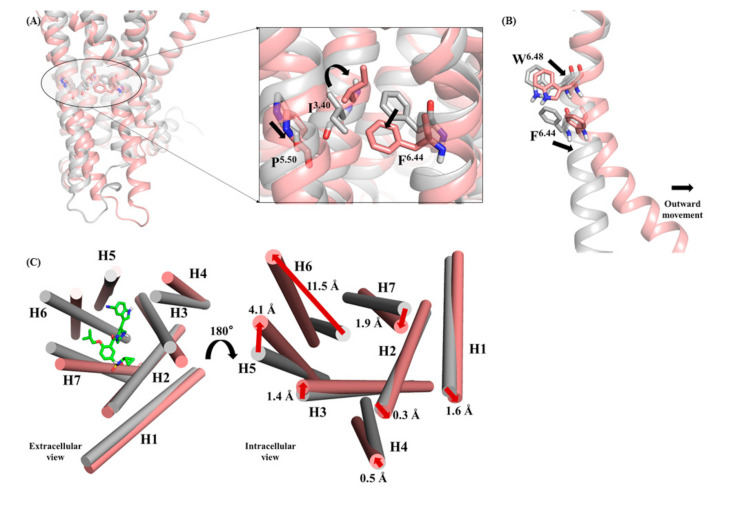
(**A**) Superimposed structures of apo and **13m**-5-HT_1A_R systems and the rearrangements of I3.40, P5.50 and F6.44 when **13m** was bound to 5-HT_1A_R. (**B**) the movement of rotamer toggle switch W6.48 in **13m**-5-HT_1A_R system contributed to the movement of the TM6. (**C**) Superimposed structures of apo and **13m**-5-HT_1A_R systems and the rearrangements of I3.40, P5.50, and F6.44 when **13m** was bound to 5-HT_1A_R. The apo system and surrounding residues are represented as grey. The **13m**-5-HT_1A_R system and surrounding residues are represented as pink.

The strong interaction between **13m** and 5-HT_1A_R induced the movement of TM3, TM5 and TM6. The triplets P5.50, I3.40 and F6.44 were located under the binding pockets of small molecules, their arrangement was important for stabilizing the antagonistic conformation of 5-HT_1A_R. The interaction of **13m** with D3.32 and T3.37 adjusted the side chain of I3.40 to flip up, subsequently leading to the relocation of P5.50 and F6.44 (Figure 5A). Their rearrangement was closely related to the movement of the helices. At the same time, W6.48 moved outward due to the steric restraints between **13m** and residue W6.48. As a rotamer toggle switch, W6.48 further pushed the intracellular end of the TM6 to move inward and the external end of the TM6 to move outward (Figure 5B). Finally, TM3, TM5 and TM6 underwent large movement to create space for the binding of G_α/s_ protein (Figure 5C). It was worth noting that the TM6 moved outward 11.5 Å.

**Figure 6:**
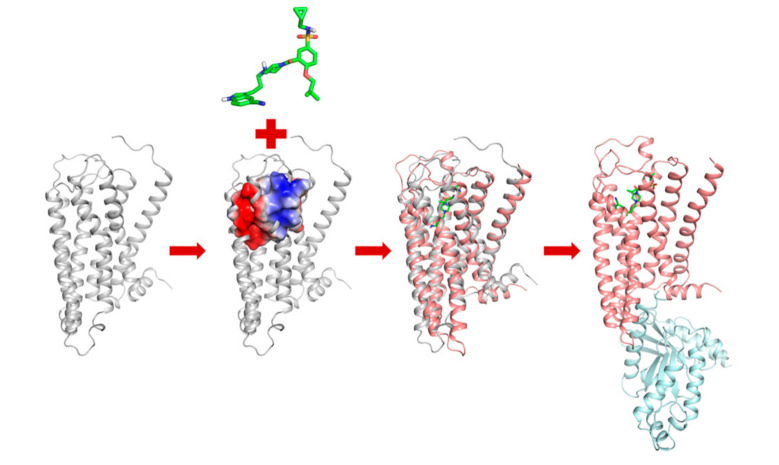
Proposed activation process of 5-HT_1A_R. The apo system and surrounding residues are represented as grey. The **13m**-5-HT_1A_R system and surrounding residues are represented as pink. The G_α/s_ protein is represented as blue.

On the basis of simulation trajectory and analysis results, a possible activation process of 5-HT_1A_R could be proposed (Figure 6). Typically, unbound 5-HT_1A_R maintained in an inactive state or a basic active state. Owing to the intramolecular interaction and inherent flexibility, the molecular thermodynamic motion would lead to the dynamic equilibrium of several energy minima conformations of 5-HT_1A_R. When **13m** got close to the binding pocket of 5-HT_1A_R, the electrostatic effect between them promoted the ligand recognition and binding process. Then, **13m** stably stayed in the pocket enclosed by TM3, TM5, TM6 and TM7 through several anchoring interactions with the residues D3.32, Q2.65 and T3.37. These residues played the role of molecular triggers, propagating the conformational changes to the inner-middle part of the seven helices. The conformational changes induced the rearrangement of P5.50, I3.40 and F6.44 as well as the adjustment of W6.48, ultimately leading to the outward movement of TM3 and TM6, along with TM5 getting closer to TM6.

The synergic helix movement of TM3, TM5, and TM6 created space for the binding of G_α/s_ protein. Then, 5-HT_1A_R was fully activated, subsequently leading to the downstream signal transduction.

## 3. Experiment Protocols

### 3.1. Chemistry and Biological Evaluation

The compound synthesis methods, NMR data and biological evaluation methods were provided in the Supplementary Materials.

### 3.2. Molecular Modeling

#### 3.2.1. Homology Modeling

The amino acid sequence of 5-HT_1A_R were downloaded from the UniProtKB database (Entry code: P08908), and sequence similarity search was performed using NCBI BLAST server (Bethesda, MD, USA) [29]. The structure (PDB code: 6G79 and 5V54) of 5-HT_1B_ receptor were selected as the templates to construct the agonistic and antagonistic conformation of 5-HT_1A_ receptor. Sequence alignment of 5-HT_1B_ receptor and 5-HT_1A_R was carried out using Discovery Studio 2016 (hereafter abbreviated to DS) (Waltham, MA, USA). Homology modeling was performed with DS. Ten models were generated after loop refinement and the one with the lowest Discrete Optimized Protein Energy (DOPE) score was submitted to energy minimization (100 steps steepest descent with backbone constrained). The PROCHECK program (Cambridge, UK) was used to evaluate the stereochemical quality of 5-HT_1A_R [30].

#### 3.2.2. Molecular Docking Operations

Molecular docking was carried out using GOLD 5.0.1 (Cambridge, UK). The binding site was defined to include all residues within a 15.0 Å radius of the conserved D3.32 Cγ carbon atom of 5-HT_1A_R. Ten conformations were produced for each ligand, and Gold-Score was used as scoring function. Other parameters were set as standard default. High-scoring complexes were inspected visually to identify the most reasonable solution.

### 3.3. Molecular Dynamic Simulations

The molecular dynamic (MD) simulations were performed using the GROMACS 5.1.2 package (Uppsala, Sweden) [31]. Two systems, **13m** bound to active-state 5-HT_1A_R and inactive-state 5-HT_1A_R without ligand (apo), were embedded in the hydrated, equilibrated palmitoyloleoylphosphatidylcholine (POPC) bilayer via the CHARMM-GUI server. The TIP3P water model was used for the MD simulation. Sodium and chloride ions were added to neutralize two systems to an ionic concentration of 0.15 mol/L. Both systems were minimized and gradually equilibrated in an NPT ensemble at 310 K and 1 bar. Periodic boundary conditions were applied to these simulation systems. Bonds connected to hydrogen atoms were restrained with the LINCS algorithm to allow an integration time step of 2 fs. Electrostatic interactions were calculated using the particle mesh Ewald (PME) method. Finally, the MD simulations for both systems were performed for 200 ns. All analysis of MD trajectories was performed using tools implemented in the GROMACS 5.1.2 package (Uppsala, Sweden). All the structural graphics were processed with PyMOL software (San Carlos, CA, USA) [32].

## 4. Conclusions

In summary, a series of indolealkylpiperazine derivatives with a benzamide moiety were designed and synthesized. Their activities on dopamine and serotonin receptors were evaluated and these compounds were found to be selective 5-HT_1A_R agonists. Among all tested compounds, **13m** displayed the most potent agonistic activity over 5-HT_1A_R with an EC_50_ value of 1.01 nM. The investigation of the binding mode uncovered the mechanism of **13m**’s potent agonistic activity and high selectivity. In addition, molecular dynamic simulations were carried out to propose **13m** induced activation model of 5-HT_1A_R. The electrostatic interaction between the negatively charged active site of the 5-HT_1A_R and the positively protonated charged **13m** is the initial driving force for the ligand recognition. **13m** formed anchoring interactions with residues D3.32, Q2.65, T3.37 and induced conformation changes of 5-HT_1A_R. This leads to the rearrangement of P5.50, I3.40 and F6.44 as well as the adjustment of W6.48, causing the outward movement of TM3 and TM6, along with TM5 getting closer to TM6. Then, 5-HT_1A_R was ready to bind G_α/s_ protein, leading itself to a fully activated state. This model would further promote structure-based agonist design of 5-HT_1A_R for the treatment of depression.

## Figures and Tables

**Table 1:**
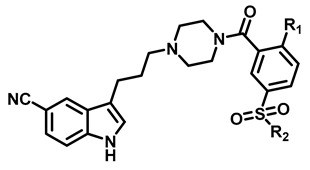
Functional activities of compounds **13a**–**13g** for dopamine receptor (D_2_R) and serotonin receptors (5-HT_1A_R and 5-HT_2A_R).

Compound	R_1_	R_2_	D_2_R	5-HT_1A_R	5-HT_2A_R
			IC_50_ (nM)	EC_50_ (nM)	IC_50_ (nM)
**FW01**	-	-	2161.35 ± 25.55 ^a^	7	206.71 ± 7.46 ^a^
**13a**	-F	-CH_3_	NA	167.7	>5000
**13b**	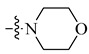	-CH_3_	NA	989.7	NA
**13c**	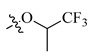	-CH_3_	NA	197.2	1144
**13d**	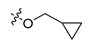	-CH_3_	NA	88.0	887.4
**13e**	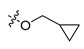	-CH_2_CH_3_	>5000	49.2	1292
**13f**	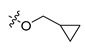	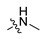	NA	23.6	1099
**13g**	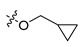	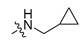	4311	5.35	2277
**Risperidone**	-	-	2.6	-	3.2
**8-OH-DPAT**	-	-	-	7.3	-

a: *K*i value; NA: no activity.

**Table 2:**
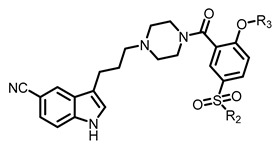
Functional activities of compounds **13f**–**13q** for dopamine receptor (D_2_R) and serotonin receptors (5-HT_1A_R and 5-HT_2A_R).

Compound	R_2_	R_3_	D_2_R	5-HT_1A_R	5-HT_2A_R
			IC_50_ (nM)	EC_50_ (nM)	IC_50_ (nM)
**13f**	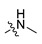	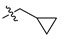	NA	23.6	1099
**13h**	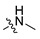	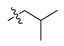	NA	5.67	567.4
**13i**	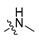	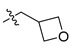	NA	400.5	4186
**13j**	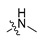	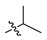	NA	NA	NA
**13k**	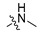	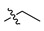	NA	77.5	1778
**13l**	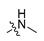	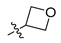	NA	523.9	NA
**13g**	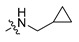	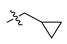	4311	5.35	2277
**13m**	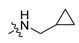	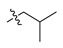	>5000	1.01	1528
**13n**	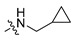	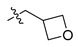	NA	31.6	>5000
**13o**	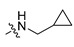	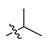	NA	22.8	NA
**13p**	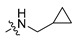	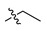	NA	48.2	4730
**13q**	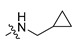	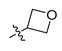	NA	174.7	NA
**Risperidone**	-	-	2.6	-	3.2
**8-OH-DPAT**	-	-	-	7.3	-

NA: no activity.

## Supplementary Materials

The following are available online, the compound synthesis methods, NMR data, biological evaluation methods.
