# Supplementary material for: Discovery of Novel Indolealkylpiperazine Derivatives as Potent 5-HT1A Receptor Agonists for the Potential Future Treatment of Depression
Source: Molecules. 2020 Nov 1;25(21):5078. doi: 10.3390/molecules25215078 (PMC7662226; doi:10.3390/molecules25215078)
Supplement: Supplementary file 1 [file molecules-25-05078-s001.pdf]

Experiment protocols

1. Chemistry

1.1. General methods and materials

Reagents and solvents were purchased from Adamas-beta and used without further purification. Analytical thin-layer chromatography was performed on HSGF 254 (0.15-0.2 mm thickness, Yantai Jiangyou Company, Yantai, Shandong, China). Column chromatography was carried out on silica gel (200-300 mesh). 1H spectra was recorded on a Brucker AMX-400/600 instrument, using TMS as an internal standard and the chemical shifts were reported in parts per million (ppm). Proton-coupling patterns were described as singlet, doublet, triplet, quartet, multiplet, and coupling constants (J) values are given in hertz (Hz). Mass spectra were given with an electric ionization (ESI) produced by HP5973N analytical mass spectrometer. All tested compounds had a minimal purity of 95% determined by HPLC.

1.2. General procedures for the preparation of compound **2**.

To a solution of aluminium chloride (9.4 g, 70.34 mmol) and 3-chloropropionyl chloride (5.04 mL, 52.76 mmol) in 100 mL anhydrous dichloromethane, a solution of 5-cyanoindole (5.00 g, 35.17 mmol) in anhydrous dichloromethane was added dropwise and then stirred at room temperature overnight. After reaction, solvent was evaporated, 100 mL ice-water was added, and then 18.8 mL concentrated hydrochloric acid was added. The aqueous phase was washed with ethyl acetate (3×20 mL), the organic phases were combined and were dried over anhydrous Na_2SO_4, filtered and evaporated. The crude product was recrystallized from ethyl acetate to give brown solid **2** (3.17 g, 19.5% yield).

1.3. General procedures for the preparation of compound **3**.

To a solution of **2** (3.17 g, 13.66 mmol) in 30 mL anhydrous tetrahydrofuran in ice-water bath was added sodium borohydride (1.03 g, 27.32 mmol). The reaction mixture was stirred at room temperature for 2 h and 30 mL trifluoroacetic acid was then added dropwise in ice-water bath. The reaction mixture was stirred at room temperature for 1 h. After reaction, the mixture was poured into ice-water and then tetrahydrofuran was evaporated. The aqueous phase was washed with ethyl acetate (3×20 mL), the organic phases were combined and dried over

anhydrous Na_2SO_4, filtered and evaporated. The residue was purified by chromatography (petroleum ether: ethyl acetate 10:1) to yield 2.65 g of **3** as white solid. (86.6% yield)

1.4. General procedures for the preparation of compound **4**.

To a solution of **3** (2.65 g, 12.15 mmol) in 30 mL acetonitrile was added potassium carbonate (2.50 g, 18.22 mmol) and boc-piperazine (3.35 g, 18.22 mmol). The reaction mixture was refluxed for 36 hours at 75 ℃. After reaction, acetonitrile was removed and 30 mL ethyl acetate was added and the organic phase was washed with water (3×20mL). The organic phases were combined and dried over anhydrous Na_2SO_4, filtered and evaporated. The residue was purified by chromatography (petroleum ether: ethyl acetate 2:1) to yield 3.55g of **4** as yellowish oily liquid. (80.4% yield)

1.5. General procedures for the preparation of compound **5**.

To a solution of **4** (3.55 g, 9.64 mmol) in dichloromethane was added trifluoroacetic acid (3.60 mL, 3.86 mmol). The reaction mixture was stirred at room temperature for 5 h. After the reaction, 70mL water was added and the aqueous phase was washed with dichloromethane (3×20 mL). The pH of the aqueous phase was then adjusted to 12 with 2M sodium hydroxide solution and the aqueous phase was again washed with dichloromethane (3×20 mL). The organic phases were combined and dried over anhydrous Na_2SO_4, filtered and evaporated. The residue could be used for the next reaction without purification.

1.6. General procedures for the preparation of compound **7**.

To a solution of chlorosulfonic acid (0.60 mol) in ice-water bath, 2-fluorobenzoic acid/2-hydroxybenzoic acid (0.15 mol) was added. The reaction mixture was stirred at 0 ℃ for 30 min and then stirred at 75℃ for 40min. After the reaction, the mixture was poured into ice-water and stirred for 30 min. After filtration, white solid **7** was obtained.

1.7. General procedures for the preparation of compound **8a**.

ⅰ) To a solution of sodium sulfite (18.80 g, 0.15 mol) in 100 mL water was added **7a** (5.00 g, 19.84 mmol) portion wise. The reaction mixture was stirred at room temperature for 2 hours, then cooled to 0 ℃ and acidified with 20% H_2SO_4. The aqueous phase was then washed with ethyl acetate (3×30 mL). The organic phases were combined and dried over anhydrous Na_2SO_4, filtered and evaporated to give 3.48 g colorless oily liquid. (81.1% yield)

ⅱ) To a solution of the product above (3.48 g, 16.00 mmol) in 30 mL *N*,*N*-dimethylformamide,

potassium carbonate (2.70 g, 19.20 mmol) was added and then iodomethane (1.2 mL, 19.20 mmol). The reaction mixture was stirred at room temperature for 3 h. After reaction, 100 mL water was added and the aqueous phase was washed with ethyl acetate (5×15 mL). The organic phases were combined and dried over anhydrous Na_2SO_4, filtered and evaporated. The residue was purified by chromatography (petroleum ether: ethyl acetate 2:1) to yield 2.52 g of **8a** as white solid. (67.8% yield)

1.8. General procedures for the preparation of compound **8b**.

ⅰ) To a solution of sodium sulfite (18.80 g, 0.15 mol) in 100 mL water was added **7b** (5.00 g, 19.95 mmol) portion wise. The reaction mixture was stirred at room temperature for 2 hours, then cooled to 0 ℃ and acidified with 20% H_2SO_4. The aqueous phase was then washed with ethyl acetate (3×30 mL). The organic phases were combined and dried over anhydrous Na_2SO_4, filtered and evaporated to give 4.04 g colorless oily liquid. (92.0% yield)

ⅱ) To a solution of the product above (4.04 g, 18.70 mmol) in 40 mL *N*,*N*-dimethylformamide, potassium carbonate (3.10 g, 22.44 mmol) was added and then iodoethane (1.80 mL, 22.44 mmol). The reaction mixture was stirred at room temperature for 3 h. After reaction, 100 mL water was added and the aqueous phase was washed with ethyl acetate (5×20 mL). The organic phases were combined and dried over anhydrous Na_2SO_4, filtered and evaporated. The residue was purified by chromatography (petroleum ether: ethyl acetate 2:1) to yield 2.10 g of **8b** as white solid. (46.0% yield)

1.9. General procedures for the preparation of compound **8c~8d**.

To a solution of **7b** (20.00 mmol) in 50 mL dichloromethane was added 25% ammonia (100.00 mmol). The reaction mixture was stirred at room temperature for 2 h. After reaction, dichloromethane was evaporated, then 100 mL water was added and the mixture was acidified with 2M hydrochloric acid. The aqueous phase was washed with ethyl acetate (3×30 mL). The organic phases were combined and dried over anhydrous Na_2SO_4, filtered and evaporated. The residue was purified by chromatography (petroleum ether: ethyl acetate 2:1) to yield **8c~8d** as white solid.

1.10. General procedures for the preparation of compound **9a~9c**.

To a solution of **8a** (0.30 g, 1.25 mmol) in 30 mL *N*,*N*-dimethylformamide, potassium carbonate (0.51 g, 3.75 mmol) was added and then corresponding reagent (1.50 mmol). The reaction

mixture was stirred at 150 ℃ for 12 h. After reaction, 30 mL water was added and the aqueous phase was washed with ethyl acetate (5×10 mL). The organic phases were combined and dried over anhydrous Na_2SO_4, filtered and evaporated. The residue was purified by chromatography (petroleum ether: ethyl acetate 2:1) to yield **9a~9c** as white solid.

1.11. General procedures for the preparation of compound **10a~10d**.

To a solution of **8a** or **9a~9c** (13.7 mmol) in 40 mL tetrahydrofuran and 10 mL water was added lithium hydroxide (25.3 mmol). The reaction mixture was stirred at room temperature for 5 hours. After reaction, tetrahydrofuran was evaporated, then water was added and the mixture was acidified with 2M hydrochloric acid. The aqueous phase was washed with ethyl acetate (3×15 mL). The organic phases were combined and dried over anhydrous Na_2SO_4, filtered and evaporated. The crude product was recrystallized from ethyl acetate to give white solid **10a~10d**.

1.12. General procedures for the preparation of compound **11a~11m**.

To a solution of **8c~8d** (2.00 mmol) in 50 mL anhydrous tetrahydrofuran was added triphenylphosphine (2.40 mmol), DBAD (2.40 mmol) and corresponding alchol (2.40 mmol). The reaction mixture was stirred at room temperature for 6 hours. After reaction, tetrahydrofuran was evaporated, then water was added and the aqueous phase was washed with ethyl acetate (3×15 mL). The organic phases were combined and dried over anhydrous Na_2SO_4, filtered and evaporated. The residue was purified by chromatography (petroleum ether: ethyl acetate 2:1) to yield **11a~1k** as colorless oily liquid.

Procedures for compounds with an oxetanyl moiety:

To a solution of **8c-d** (2.04 mmol) in 50 mL *N*,*N*-dimethylformamide was added cesium carbonate (3.06 mmol) and 3-iodo-oxetane (3.06 mmol). The reaction mixture was stirred at 80 ℃ for 10 hours. After reaction, water was added and the aqueous phase was washed with ethyl acetate (5×15 mL). The organic phases were combined and dried over anhydrous Na_2SO_4, filtered and evaporated. The residue was purified by chromatography (petroleum ether: ethyl acetate 1:2) to yield **11l~11m** as colorless oily liquid.

1.13. General procedures for the preparation of compound **12a~12m**.

To a solution of **11a~11m** (2.00 mmol) in 10 mL tetrahydrofuran and 10 mL water was added lithium hydroxide (4.00 mmol). The reaction mixture was stirred at room temperature for 5 h.

After reaction, tetrahydrofuran was evaporated, then water was added and the mixture was acidified with 2M hydrochloric acid. The aqueous phase was washed with ethyl acetate (3×15 mL). The organic phases were combined and dried over anhydrous Na_2SO_4, filtered and evaporated. The crude product was recrystallized from ethyl acetate to give white solid **12a~12m**.

1.14. General procedures for the preparation of compound **13a~13q**.

To a solution of **10a~10d** or **12a~12m** (1.50 mmol) in 20 mL dichloromethane was added EDC•HCl (2.20 mmol), DBAB (2.20 mmol) and *N*,*N*-diisopropylethylamine (3.00 mmol). The reaction mixture was stirred at room temperature for 1 h and then **5** (1.80 mmol) was added. The reaction mixture was stirred at room temperature for further 8 h. After reaction, 30 mL water was added and the mixture was washed with dichloromethane (3×15 mL). The organic phases were combined and dried over anhydrous Na_2SO_4, filtered and evaporated. The residue was purified by chromatography (dichloromethane: methanol 40:1) to yield **13a~13q** as colorless oily liquid.

1.14.1. 3-(3-(4-(2-fluoro-5-(methylsulfonyl)benzoyl)piperazin-1-yl)propyl)-1*H*-indole-5-carbonitrile **13a**. Compound **13a** was prepared from 2-fluoro-5-(methylsulfonyl)benzoic acid (**10a**) and 3-(3-(piperazin-1-yl)propyl)-1*H*-indole-5-carbonitrile (**5**) as colorless oily liquid. Yield 71.1%. ^{1}H NMR (400 MHz, DMSO-d_6) δ 11.40 (s, 1H), 8.09 (s, 2H), 7.71 – 7.58 (m, 1H), 7.51 – 7.47 (m, 1H), 7.40 (d, *J* = 8.7 Hz, 1H), 7.35 (s, 1H), 3.68 (s, 2H), 3.25 (s, 2H), 3.17 (s, 2H), 2.74 (d, *J* = 7.7 Hz, 2H), 2.40 – 2.28 (m, 4H), 1.80 (s, 2H), 1.24 (s, 2H). ^{13}C NMR (151 MHz, DMSO-d_6) δ 206.59, 162.28, 138.08, 137.87, 130.86, 129.79, 128.55, 127.26, 125.12, 124.40, 123.70, 121.08, 117.43, 115.90, 112.71, 100.30, 57.23, 53.00, 46.83, 43.53, 27.19, 22.03. HRMS (ESI$^+$) m/z $[M+H]^+$ calculated: 469.1704, found: 469.1696.

1.14.2. 3-(3-(4-(5-(methylsulfonyl)-2-morpholinobenzoyl)piperazin-1-yl)propyl)-1H-indole-5-carbonitrile **13b**. Compound **13b** was prepared from 5-(methylsulfonyl)-2-morpholinobenzoic acid (**10b**) and 3-(3-(piperazin-1-yl)propyl)-1*H*-indole-5-carbonitrile (**5**) as colorless oily liquid. Yield 70.59%. ^{1}H NMR (400 MHz, $CDCl_3$) δ 8.46 (s, 1H), 7.96 (s, 1H), 7.91–7.84 (m, 1H), 7.82-7.93 (m, 1H), 7.39 (s, 2H), 7.13–7.03 (m, 2H), 4.11 (s, 1H), 3.80 (s, 3H), 3.53 (s, 1H), 3.33 (s, 3H), 3.17 (d, *J* = 13.2 Hz, 1H), 3.03 (s, 6H), 2.79 (t, *J* = 7.2 Hz, 2H), 2.67 (s, 1H), 2.48-2.40 (m, 4H), 2.19 (s, 1H), 1.89 (s, 2H). ^{13}C NMR (100 MHz, $CDCl_3$) δ 165.24, 155.64, 139.34, 134.34, 129.90, 128.95,

128.39, 126.08, 124.22, 123.55, 118.82, 114.27, 113.43, 112.77, 101.83, 65.85, 56.23, 52.34, 47.41, 44.58, 43.80, 26.93, 24.93. HRMS (ESI$^+$) m/z [M+H]$^+$ calculated: 536.2326, found: 536.2337.

1.14.3 3-(3-(4-(5-(methylsulfonyl)-2-((1,1,1-trifluoropropan-2-yl)oxy)benzoyl)piperazin-1-yl)propyl)-1H-indole-5-carbonitrile **13c**. Compound **13c** was prepared from 5-(methylsulfonyl)-2-((1,1,1-trifluoropropan-2-yl)oxy)benzoic acid (**10c**) and 3-(3-(piperazin-1-yl)propyl)-1*H*-indole-5-carbonitrile (**5**) as colorless oily liquid. Yield 46.7%. ^{1}H NMR (400 MHz, $CDCl_3$) δ 8.41 (s, 1H), 7.98–7.85 (m, 3H), 7.40 (s, 2H), 7.09-6.92 (m, 2H), 4.81-4.73 (m, 1H), 3.83-3.75 (m, 2H), 3.25 (s, 2H), 3.05 (s, 3H), 2.79 (t, *J* = 7.2 Hz, 2H), 2.63- 2.25 (m, 6H), 1.90 (s, 2H), 1.57 (d, *J* = 6.4 Hz, 1H), 1.51 (d, *J* = 8.4 Hz, 2H). ^{13}C NMR (100 MHz, $CDCl_3$) δ 164.91, 155.40, 139.34, 134.34, 131.40, 130.37, 128.57, 126.08, 123.19, 121.62, 121.07, 113.43, 113.10, 112.77, 101.83, 73.09, 56.23, 52.34, 44.58, 43.80, 26.93, 24.93, 15.76. HRMS (ESI$^+$) m/z [M+H]$^+$ calculated: 563.1934, found: 563.1944.

1.14.4. 3-(3-(4-(2-(cyclopropylmethoxy)-5-(methylsulfonyl)benzoyl)piperazin-1-yl)propyl)-1*H*-indole-5-carbonitrile **13d**. Compound **13d** was prepared from 2-(cyclopropylmethoxy)-5-(methylsulfonyl)benzoic acid (**10d**) and 3-(3-(piperazin-1-yl)propyl)-1*H*-indole-5-carbonitrile (**5**) as colorless oily liquid. Yield 43.7%.^{1}H NMR (400 MHz, DMSO-d_6) δ 11.41 (s, 1H), 8.08 (s, 1H), 7.90 (d, *J* = 8.8 Hz, 1H), 7.70 (s, 1H), 7.49 (d, *J* = 8.4 Hz, 1H), 7.39 (d, *J* = 8.6 Hz, 1H), 7.34 (s, 1H), 7.28 (d, *J* = 8.9 Hz, 1H), 4.07 – 3.93 (m, 2H), 3.64 (d, *J* = 42.4 Hz, 2H), 2.73 (s, 2H), 2.34 (s, 4H), 1.92 (s, 1H), 1.80 (s, 2H), 1.23 (s, 4H), 0.56 (d, *J* = 7.8 Hz, 2H), 0.33 (d, *J* = 4.7 Hz, 2H). ^{13}C NMR (151 MHz, DMSO-d_6) δ 206.59, 164.85, 158.08, 138.09, 132.78, 129.90, 127.25, 126.64, 125.14, 124.38, 123.70, 121.07, 115.90, 112.91, 112.72, 100.31, 73.16, 57.35, 52.57, 43.93, 40.24, 30.83, 22.02, 9.98, 3.19. HRMS (ESI$^+$) m/z [M+H]$^+$ calculated: 521.2217, found: 527.2213.

1.14.5. 3-(3-(4-(2-(cyclopropylmethoxy)-5-(ethylsulfonyl)benzoyl)piperazin-1-yl)propyl)-1*H*-indole-5-carbonitrile **13e**. Compound **13e** was prepared from 2-(cyclopropylmethoxy)-5-(ethylsulfonyl)benzoic acid (**12a**) and 3-(3-(piperazin-1-yl)propyl)-1*H*-indole-5-carbonitrile (**5**) as colorless oily liquid. Yield 43.7%. ^{1}H NMR (400 MHz, DMSO-d_6) *δ* 11.40(s, 1H), 8.09(s, 1H), 7.86(d, *J*=8.0 Hz, 1H), 7.63(s, 1H), 7.49(d, *J*=12.0 Hz, 1H), 7.40(d, *J*=12.0 Hz, 1H), 7.34(s, 1H), 7.29(d, *J*=8.0 Hz, 1H), 3.95-4.03(m, 2H), 3.65(d, *J*=48.0 Hz, 2H), 3.28(q, *J*=8.0 Hz, 2H), 3.15(s, 2H), 2.72(t, *J*=8.0 Hz, 2H), 2.32-2.46(m, 6H), 1.78-1.81(m, 2H), 1.23(s, 1H), 1.08(t, *J*=8.0 Hz, 3H), 0.56(d, *J*=8.0 Hz, 2H), 0.33(d, *J*=4.0 Hz, 2H). ^{13}C NMR (151 MHz, DMSO-d_6) δ 206.58, 164.79, 158.24,

138.08, 130.73, 130.23, 127.75, 127.27, 126.74, 125.11, 124.38, 123.69, 121.07, 115.91, 112.70, 100.30, 73.14, 57.34, 53.00, 49.52, 46.56, 27.20, 22.03, 9.97, 7.39, 3.42. HRMS (ESI$^+$) m/z [M+H]$^+$ calculated: 535.2374, found: 535.2370.

1.14.6. 3-(4-(3-(5-cyano-1*H*-indol-3-yl)propyl)piperazine-1-carbonyl)-4-(cyclopropylmethoxy)-*N*-methylbenzenesulfonamide **13f**. Compound **13f** was prepared from 2-(cyclopropylmethoxy)-5-(*N*-methylsulfamoyl)benzoic acid (**12b**) and 3-(3-(piperazin-1-yl)propyl)-1*H*-indole-5-carbonitrile (**5**) as colorless oily liquid. Yield 51.1%. ^{1}H NMR (400 MHz, DMSO-d_6) δ 11.40(s, 1H), 8.09(s, 1H), 7.75(d, *J*=8.0 Hz, 1H), 7.53(d, *J*=4.0 Hz, 1H), 7.49(d, *J*=12.0 Hz, 1H), 7.37-7.41(m, 2H), 7.34(s, 1H), 7.24(d, *J*=8.0 Hz, 1H), 3.92-4.00(m, 2H), 3.65(d, *J*=18.0 Hz, 2H), 3.16(s, 2H), 2.72(t, *J*=7.2 Hz, 2H), 2.34-2.40(m, 9H), 1.79(p, *J*=8.0 Hz, 2H), 1.21(s, 1H), 0.56(m, *J*=8.0 Hz, 2H), 0.33(d, *J*=8.0 Hz, 2H). ^{13}C NMR (151 MHz, DMSO-d_6) δ 206.58, 165.03, 157.19, 138.08, 131.09, 129.54, 127.27, 126.65, 126.49, 125.11, 124.38, 123.69, 121.07, 115.91, 112.75, 100.30, 73.00, 57.35, 53.04, 46.56, 28.81, 27.20, 22.03, 10.01, 3.41. HRMS (ESI$^+$) m/z [M+H]$^+$ calculated: 535.2326, found: 535.2322.

1.14.7. 3-(4-(3-(5-cyano-1*H*-indol-3-yl)propyl)piperazine-1-carbonyl)-4-(cyclopropylmethoxy)-*N*-(cyclopropylmethyl)benzenesulfonamide **13g**. Compound **13g** was prepared from 2-(cyclopropylmethoxy)-5-(*N*-(cyclopropylmethyl)sulfamoyl)benzoic acid (**12c**) and 3-(3-(piperazin-1-yl)propyl)-1*H*-indole-5-carbonitrile (**5**) as colorless oily liquid. Yield 46.6%. ^{1}H NMR (400 MHz, DMSO-d_6) δ 11.40 (s, 1H), 8.09(s, 1H), 7.76(d, *J*=8.0 Hz, 1H), 7.65(t, *J*=6.0 Hz, 1H), 7.56(s, 1H), 7.49(d, *J*=8.0 Hz, 1H), 7.40(d, *J*=8.0 Hz, 1H), 7.34 (s, 1H), 7.21(d, *J*=8.0 Hz, 1H), 3.89-4.02(dt, *J*=32.0, 8.0 Hz, 2H), 3.64(d, *J*=16.0 Hz, 2H), 3.14(d, *J*=12.0 Hz, 2H), 2.72(t, *J*=4.0 Hz, 2H), 2.61(t, *J*=6.4 Hz, 2H), 2.28-2.40(m, 6H), 1.78-1.81(m, 2H), 0.84-0.87(m, 1H), 0.74-0.77(m, 1H), 0.55(d, *J*=8.0 Hz, 2H), 0.34(d, *J*=4.0 Hz, 4H), 0.06(d, *J*=4.0 Hz, 2H). ^{13}C NMR (151 MHz, DMSO-d_6) δ 206.58, 165.08, 157.01, 138.07, 132.93, 129.24, 127.27, 126.47, 125.11, 124.38, 123.68, 121.06, 115.90, 112.70, 112.62, 100.30, 72.96, 57.36, 53.03, 47.44, 46.54, 27.19, 22.03, 10.75, 10.01, 3.58, 3.17. HRMS (ESI$^+$) m/z [M+H]$^+$ calculated: 576.2639, found: 576.2645.

1.14.8. 3-(4-(3-(5-cyano-1*H*-indol-3-yl)propyl)piperazine-1-carbonyl)-4-isobutoxy-*N*-methylbenzenesulfonamide **13h**. Compound **13h** was prepared from 2-isobutoxy-5-(*N*-methylsulfamoyl)benzoic acid (**12d**) and 3-(3-(piperazin-1-yl)propyl)-1*H*-indole-5-carbonitrile (**5**) as colorless oily liquid. Yield 48.4%. ^{1}H NMR (400 MHz, DMSO-d_6) δ 11.39(s, 1H), 8.08(s,

1H), 7.76(dd, *J*=8.0, 4.0 Hz, 1H), 7.65(t, *J*=6.0 Hz, 1H), 7.54(d, *J*=4.0 Hz, 1H), 7.49(d, *J*=8.0 Hz, 1H), 7.40(d, *J*=8.0 Hz, 1H), 7.34(s, 1H), 7.26(d, *J*=8.0 Hz, 1H), 4.77(p, *J*=6.4 Hz, 2H), 3.63(s, 2H), 3.11(s, 2H), 2.72(t, *J*=8.0 Hz, 2H), 2.62(t, *J*=6.4 Hz, 2H), 2.40(s, 2H), 2.26-2.35(m, 4H), 1.78(q, *J*=8.0 Hz, 2H), 1.27(dd, *J*=12.0, 8.0 Hz, 6H), 0.75-0.79(m, 1H), 0.31-0.36(m, 2H), 0.04-0.08(m, 2H). ^{13}C NMR (151 MHz, DMSO-d_6) δ 206.59, 165.04, 157.39, 138.07, 131.13, 129.57, 127.27, 126.55, 126.29, 125.11, 124.38, 123.69, 121.06, 115.90, 112.74, 100.30, 74.59, 57.32, 52.98, 46.51, 28.81, 27.89, 27.15, 22.02, 19.05. HRMS (ESI$^+$) m/z [M+H]$^+$ calculated: 538.2483, found: 538.2481.

4.1.14.9. 3-(4-(3-(5-cyano-1*H*-indol-3-yl)propyl)piperazine-1-carbonyl)-*N*-methyl-4-(oxetan-3-ylmethoxy)benzenesulfonamide **13i**. Compound **13i** was prepared from 5-(*N*-methylsulfamoyl)-2-(oxetan-3-ylmethoxy)benzoic acid (**12e**) and 3-(3-(piperazin-1-yl)propyl)-1*H*-indole-5-carbonitrile (**5**) as colorless oily liquid. Yield 43.8%. ^{1}H NMR (400 MHz, DMSO-d_6) *δ* 11.40(s, 1H), 8.09(s, 1H), 7.79(dd, *J*=8.0, 4.0 Hz, 1H), 7.54(t, *J*=2.0 Hz, 1H), 7.49(d, *J*=12.0 Hz, 1H), 7.38-7.42(m, 2H), 7.34(s, 1H), 7.32(s, 1H), 4.67(t, *J*=8.0 Hz, 2H), 4.26-4.43(m, 4H), 3.62(s, 2H), 3.38-3.43(m, 1H), 3.14(s, 2H), 2.72(t, *J*=8.0 Hz, 2H), 2.27-2.40(m, 9H), 1.79(p, *J*=8.0 Hz, 2H). ^{13}C NMR (151 MHz, DMSO-d_6) δ 206.58, 164.85, 157.07, 138.07, 131.56, 129.55, 127.27, 126.46, 125.10, 124.39, 123.69, 121.07, 115.91, 113.02, 112.70, 100.30, 73.06, 69.69, 57.31, 53.11, 46.49, 33.89, 28.82, 27.19, 22.02. HRMS (ESI$^+$) m/z [M+H]$^+$ calculated: 552.2275, found: 552.2271.

1.14.10. 3-(4-(3-(5-cyano-1*H*-indol-3-yl)propyl)piperazine-1-carbonyl)-4-isopropoxy-*N*-methylbenzenesulfonamide **13j**. Compound **13j** was prepared from 2-isopropoxy-5-(*N*-methylsulfamoyl)benzoic acid (**12f**) and 3-(3-(piperazin-1-yl)propyl)-1*H*-indole-5-carbonitrile (**5**) as colorless oily liquid. Yield 44.6%. ^{1}H NMR (400 MHz, DMSO-d_6) *δ* 11.41(s, 1H), 8.08(s, 1H), 7.70(d, *J*=8.0 Hz, 1H), 7.47-7.51(m, 2H), 7.41(d, *J*=8.0 Hz, 1H), 7.36(s, 1H), 7.26(d, *J*=12.0 Hz, 1H), 4.78(p, *J*=6.0 Hz, 1H), 3.53(d, *J*=14.0 Hz, 2H), 2.99(t, *J*=6.4 Hz, 4H), 2.59-2.72(m, 9H), 1.80(p, *J*=8.0 Hz, 2H), 1.26-1.30(m, 6H). ^{13}C NMR (151 MHz, DMSO-d_6) δ 206.58, 164.97, 156.45, 138.10, 129.79, 128.43, 127.42, 127.15, 125.24, 124.35, 123.77, 121.07, 115.25, 113.49, 112.74, 100.39, 70.82, 49.58, 47.51, 45.82, 34.74, 27.83, 21.76, 21.47. HRMS (ESI$^+$) m/z [M+H]$^+$ calculated: 524.2326, found: 524.2325.

1.14.11. 3-(4-(3-(5-cyano-1*H*-indol-3-yl)propyl)piperazine-1-carbonyl)-4-ethoxy-*N*-methylbenzenesulfonamide **13k**. Compound **13k** was prepared from 2-ethoxy-5-(*N*-methylsulfamoyl)benzoic acid (**12g**) and 3-(3-(piperazin-1-yl)propyl)-1*H*-indole-5-carbonitrile

(**5**) as colorless oily liquid. Yield 31.6%. ^{1}H NMR (400 MHz, DMSO-d_6) δ 11.40(s, 1H), 8.09(s, 1H), 7.76(dd, *J*=8.0, 4.0 Hz, 1H), 7.53(d, *J*=4.0 Hz, 2H), 7.48(d, *J*=8.0 Hz, 1H), 7.37-7.40(m, 2H), 7.34(d, *J*=2.4 Hz, 1H), 7.27(d, *J*=8.0 Hz, 1H), 4.17(q, *J*=8.0 Hz, 2H), 3.64(s, 2H), 3.14(s, 2H), 2.72(t, *J*=8.0 Hz, 2H), 2.31-2.40(m, 9H), 1.80(q, *J*=8.0 Hz, 2H), 1.33(t, *J*=6.8 Hz, 3H). ^{13}C NMR (151 MHz, DMSO-d_6) δ 206.58, 165.01, 157.08, 138.07, 131.15, 129.60, 127.28, 126.65, 126.45, 125.11, 124.40, 123.69, 121.07, 115.91, 112.61, 100.30, 64.39, 57.34, 53.08, 46.49, 28.81, 27.19, 22.02, 14.59. HRMS (ESI$^+$) m/z [M+H]$^+$ calculated: 510.2170, found: 510.2168.

4.1.14.12. 3-(4-(3-(5-cyano-1*H*-indol-3-yl)propyl)piperazine-1-carbonyl)-*N*-methyl-4-(oxetan-3-yloxy)benzenesulfonamide **13l**. Compound **13l** was prepared from 5-(*N*-methylsulfamoyl)-2-(oxetan-3-yloxy)benzoic acid (**12h**) and 3-(3-(piperazin-1-yl)propyl)-1*H*-indole-5-carbonitrile (**5**) as colorless oily liquid. Yield 38.7%. ^{1}H NMR (400 MHz, DMSO-d_6) δ 11.39(s, 1H), 8.09(s, 1H), 7.74(d, *J*=12.0 Hz, 1H), 7.60(s, 1H), 7.49(d, *J*=8.0 Hz, 1H), 7.41(s, 1H), 7.34(s, 1H), 6.91(d, *J*=8.0 Hz, 1H), 5.43(s, 1H), 4.96(s, 2H), 4.54(s, 2H), 3.67(d, *J*=52.0 Hz, 2H), 3.26(d, *J*=16.0 Hz, 2H), 2.72(t, *J*=8.0 Hz, 4H), 2.35-2.46(m, 9H), 1.80(t, *J*=4.0 Hz, 2H). ^{13}C NMR (151 MHz, DMSO-d_6) δ 206.59, 164.60, 154.81, 138.08, 132.28, 129.60, 127.27, 127.07, 126.68, 125.10, 124.40, 123.70, 121.07, 115.91, 112.70, 100.30, 76.66, 71.05, 57.37, 53.09, 46.58, 28.82, 27.15, 22.04. HRMS (ESI$^+$) m/z [M+H]$^+$ calculated: 538.2119, found: 538.2114.

1.14.13. 3-(4-(3-(5-cyano-1*H*-indol-3-yl)propyl)piperazine-1-carbonyl)-*N*-(cyclopropylmethyl)-4-isobutoxybenzenesulfonamide **13m**. Compound **13m** was prepared from 5-(*N*-(cyclopropylmethyl)sulfamoyl)-2-isobutoxybenzoic acid (**12i**) and 3-(3-(piperazin-1-yl)propyl)-1*H*-indole-5-carbonitrile (**5**) as colorless oily liquid. Yield 47.8%. ^{1}H NMR (400 MHz, DMSO-d_6) δ 11.40 (s, 1H), 8.08(s, 1H), 7.76-7.78(dd, *J*=8.0, 2.0 Hz, 1H), 7.65(t, *J*=5.6 Hz, 1H), 7.55(d, *J*=2.4 Hz, 1H), 7.49(d, *J*=12.0 Hz, 1H), 7.40(dd, *J*=8.0, 4.0 Hz, 1H), 7.33(d, *J*=4.0 Hz, 1H), 7.24(d, *J*=8.0 Hz, 1H), 3.81-3.91(m, 2H), 3.64(d, *J*=40.0 Hz, 2H), 3.12(s, 2H), 2.72(t, *J*=7.2 Hz, 2H), 2.61(t, *J*=6.4 Hz, 2H), 2.41(s, 2H), 2.23-2.34(m, 4H), 2.00(p, *J*=6.4 Hz, 1H), 1.78(p, *J*=7.2 Hz, 2H), 0.95(d, *J*=4.0 Hz, 6H), 0.73-0.79(m, 1H), 0.31-0.36(m, 2H), 0.05-0.08(m, 2H). ^{13}C NMR (151 MHz, DMSO-d_6) δ 206.58, 165.09, 157.21, 138.07, 132.95, 129.27, 127.27, 126.46, 126.13, 125.11, 124.37, 123.69, 121.06, 115.90, 112.70, 100.30, 74.56, 57.33, 52.96, 47.45, 46.49, 27.89, 27.15, 22.02, 19.04, 10.75, 3.56. HRMS (ESI$^+$) m/z [M+H]$^+$ calculated: 578.2796, found: 578.2784.

1.14.14. 3-(4-(3-(5-cyano-1*H*-indol-3-yl)propyl)piperazine-1-carbonyl)-*N*-(cyclopropylmethyl)-

4-(oxetan-3-ylmethoxy)benzenesulfonamide **13n**. Compound **13n** was prepared from 5-(*N*-(cyclopropylmethyl)sulfamoyl)-2-(oxetan-3-ylmethoxy)benzoic acid (**12j**) and 3-(3-(piperazin-1-yl)propyl)-1*H*-indole-5-carbonitrile (**5**) as colorless oily liquid. Yield 50.3%. ^{1}H NMR (400 MHz, DMSO-d_6) δ 11.40 (s, 1H), 8.09(s, 1H), 7.77-7.80(dd, *J*=8.0, 2.0 Hz, 1H), 7.68(t, *J*=6.0 Hz, 1H), 7.57(d, *J*=2.4 Hz, 1H), 7.49(d, *J*=8.0 Hz, 1H), 7.40(dd, *J*=8.0, 4.0 Hz, 1H), 7.34(d, *J*=5.2 Hz, 1H), 7.30(d, *J*=8.0 Hz, 1H), 4.66-4.70(m, 2H), 4.42(q, *J*=4.0 Hz, 2H), 4.25-4.38(m, 2H), 3.62(d, *J*=16.0 Hz, 2H), 3.40(q, *J*=8.0 Hz, 1H), 3.12(t, *J*=4.0 Hz, 2H), 2.72(t, *J*=8.0 Hz, 2H), 2.63(t, *J*=6.4 Hz, 2H), 2.39(t, *J*=4.0 Hz, 2H), 2.33(t, *J*=6.8 Hz, 2H), 2.25(d, *J*=8.0 Hz, 2H), 1.79(p, *J*=8.0 Hz, 2H), 0.74-0.81(m, 1H), 0.32-0.36(m, 2H), 0.05-0.08(m, 2H). ^{13}C NMR (151 MHz, DMSO-d_6) δ 206.58, 164.89, 156.89, 138.07, 133.39, 129.26, 127.27, 126.61, 126.29, 125.10, 124.39, 123.69, 121.07, 115.90, 112.70, 100.30, 73.06, 69.67, 57.31, 53.09, 47.45, 46.48, 33.89, 27.18, 22.02, 10.76, 3.57. HRMS (ESI$^+$) m/z [M+H]$^+$ calculated: 592.2588, found: 592.2580.

1.14.15. 3-(4-(3-(5-cyano-1*H*-indol-3-yl)propyl)piperazine-1-carbonyl)-*N*-(cyclopropylmethyl)-4-isopropoxybenzenesulfonamide **13o**. Compound **13o** was prepared from 5-(*N*-(cyclopropylmethyl)sulfamoyl)-2-isopropoxybenzoic acid (**12k**) and 3-(3-(piperazin-1-yl)propyl)-1*H*-indole-5-carbonitrile (**5**) as colorless oily liquid. Yield 43.8%. ^{1}H NMR (400 MHz, DMSO-d_6) δ 11.40 (s, 1H), 8.09(s, 1H), 7.74-7.77(dd, *J*=8.0, 4.0 Hz, 1H), 7.65(t, *J*=6.0 Hz, 1H), 7.54(d, *J*=4.0 Hz, 1H), 7.49(d, *J*=8.0 Hz, 1H), 7.40(d, *J*=8.0 Hz, 1H), 7.34(s, 1H), 7.26(d, *J*=8.0 Hz, 1H), 4.77(p, *J*=6.0 Hz, 1H), 3.63(s, 2H), 3.11(s, 2H), 2.72(t, *J*=8.0 Hz, 2H), 2.62(t, *J*=6.4 Hz, 2H), 2.40(s, 2H), 2.26-2.35(m, 4H), 1.78(q, *J*=8.0 Hz, 2H), 1.25-1.30(dd, *J*=12.0, 8.0 Hz, 6H), 0.75-0.79(m, 1H), 0.31-0.36(m, 2H), 0.04-0.08(m, 2H). ^{13}C NMR (151 MHz, DMSO-d_6) δ 204.58, 163.17, 153.90, 136.08, 130.71, 127.15, 125.27, 124.68, 123.11, 122.39, 121.68, 119.06, 113.90, 111.36, 110.70, 98.30, 68.71, 55.37, 51.10, 45.43, 44.44, 25.15, 20.03, 19.71, 8.77, 1.60. HRMS (ESI$^+$) m/z [M+H]$^+$ calculated: 564.2639, found: 564.2635.

1.14.16. 3-(4-(3-(5-cyano-1*H*-indol-3-yl)propyl)piperazine-1-carbonyl)-*N*-(cyclopropylmethyl)-4-ethoxybenzenesulfonamide **13p**. Compound **13p** was prepared from 5-(*N*-(cyclopropylmethyl)sulfamoyl)-2-ethoxybenzoic acid (**12l**) and 3-(3-(piperazin-1-yl)propyl)-1*H*-indole-5-carbonitrile (**5**) as colorless oily liquid. Yield 40.1%. ^{1}H NMR (400 MHz, DMSO-d_6) δ 11.40 (s, 1H), 8.09(s, 1H), 7.78(dd, *J*=8.0, 4.0 Hz, 1H), 7.66(t, *J*=6.0 Hz, 1H), 7.55(d, *J*=4.0 Hz, 1H), 7.48(d, *J*=12.0 Hz, 1H), 7.40(d, *J*=8.0 Hz, 1H), 7.34(s, 1H), 7.24(d, *J*=8.0 Hz, 1H), 4.16(q, *J*=6.4 Hz,

2H), 3.63(s, 2H), 3.12(s, 2H), 2.72(t, *J*=8.0 Hz, 2H), 2.62(t, *J*=6.0 Hz, 2H), 2.40(s, 2H), 2.26-2.35(m, 4H), 1.79(p, *J*=8.0 Hz, 2H), 1.33(t, *J*=7.2 Hz, 3H), 0.73-0.79(m, 1H), 0.33(d, *J*=4.0 Hz, 2H), 0.06(d, *J*=4.0 Hz, 2H). ^{13}C NMR (151 MHz, DMSO-d_6) δ 206.58, 165.05, 156.90, 138.07, 132.98, 129.30, 127.27, 126.47, 125.11, 124.39, 123.68, 121.06, 115.90, 112.70, 112.48, 100.29, 64.35, 57.34, 53.06, 47.44, 46.47, 27.19, 22.02, 14.59, 10.76, 3.57. HRMS (ESI$^+$) m/z [M+H]$^+$ calculated: 550.2483, found: 550.2480.

1.14.17. 3-(4-(3-(5-cyano-1*H*-indol-3-yl)propyl)piperazine-1-carbonyl)-*N*-(cyclopropylmethyl)-4-(oxetan-3-yloxy)benzenesulfonamide **13q**. Compound **13q** was prepared from 5-(*N*-(cyclopropylmethyl)sulfamoyl)-2-(oxetan-3-yloxy)benzoic acid (**12m**) and 3-(3-(piperazin-1-yl)propyl)-1*H*-indole-5-carbonitrile (**5**) as colorless oily liquid. Yield 38.5%. ^{1}H NMR (400 MHz, DMSO-d_6) δ 11.40 (s, 1H), 8.09(s, 1H), 7.70-7.75(m, 2H), 7.62(d, *J*=1.6 Hz, 1H), 7.49(d, *J*=12.0 Hz, 1H), 7.40(d, *J*=8.0 Hz, 1H), 7.34(s, 1H), 6.88(d, *J*=8.0 Hz, 1H), 5.39-5.44(m, 1H), 4.95(q, *J*=8.0 Hz, 2H), 4.50-4.57(m, 2H), 3.67(d, *J*=32.0 Hz, 2H), 3.18(d, *J*=20.0 Hz, 2H), 2.73(t, *J*=7.2 Hz, 2H), 2.63(t, *J*=6.4 Hz, 2H), 2.44(s, 2H), 2.34(t, *J*=7.2 Hz, 4H), 1.76-1,83(m, 2H), 0.77(s, 1H), 0.33-0.36(m, 2H), 0.06-0.08(m, 2H). ^{13}C NMR (151 MHz, DMSO-d_6) δ 206.58, 164.65, 154.63, 138.08, 134.10, 129.30, 127.27, 126.89, 126.58, 125.10, 124.40, 123.69, 121.07, 115.91, 112.70, 100.30, 76.56, 71.02, 57.37, 53.08, 47.43, 46.56, 27.15, 22.04, 10.79, 3.61. HRMS (ESI$^+$) m/z [M+H]$^+$ calculated: 578.2432, found: 578.2422.

1.2 Biological Evaluation

1.2.1. Ultra Lance cAMP assay

The Ultra Lance cAMP assay was performed to evaluate the function of synthesized compounds to D_2R and 5-$HT_{1A}R$ with human D_2R and 5-$HT_{1A}R$ expressed by HEK-293 cells. Reference compounds for D_2R and 5-$HT_{1A}R$ were risperidone and 8-OH-DPAT. Procedures for Ultra Lance cAMP assay: 1) Transfer compound to assay plate by Echo (compound total volume 100 nl); 2) Collect cells with stimulation buffer; 3) Transfer 10 µl of cell solution to assay plate; 4) Centrifuge at 600 rpm for 3 min and incubate 60 min at room temperature; 5) Add 5 µl 4X Eu-cAMP tracer solution and 5 µl 4X ULight™-anti-cAMP solution to assay plate; 6) Centrifuge at 600 rpm for 3 min and incubate 60 min at room temperature; 7) Read plate on EnVision. The IC_{50} values were calculated by nonlinear regression using a sigmoidal function.

1.2.2. Fluorometric Imaging Plate Reader (FLIPR) assay

The FLIPR assay was performed to evaluate the function of synthesized compounds to 5-HT_{2A}R with human 5-HT_{2A}R expressed by CHO-K1 cells. Reference compound for 5-HT_{2A}R was risperidone. Procedures for FLIPR assay: 1) Add 50 μl of the cell suspension to each well on the assay plate and place the assay plate in 37 ℃, 5% CO_2 incubate for 16-24 h; 2) Remove cell plates from the incubator or centrifuge, remove the supernatant and add 30 μl 1* dye; 3) Place the assay plates in the 37 ℃, 5% CO_2 incubator for 1 h; 4) add 30 μl assay buffer into the assay plate and then shake for 20-40 min; 5) Place the plates on the FLIPR and 15 μl /well of test compound is added and calcium flux signal is measured. After 15 min add 22.5 μl /well of inducer and calcium flux signal is measured. The IC_{50} values were calculated by nonlinear regression using a sigmoidal function.
